# Effects of Ambient Air Pollution on Hemostasis and Inflammation

**DOI:** 10.1289/ehp.0800437

**Published:** 2009-02-22

**Authors:** Goran Rudež, Nicole A.H. Janssen, Evren Kilinc, Frank W.G. Leebeek, Miriam E. Gerlofs-Nijland, Henri M.H. Spronk, Hugo ten Cate, Flemming R. Cassee, Moniek P.M. de Maat

**Affiliations:** 1 Department of Hematology, Erasmus University Medical Center, Rotterdam, the Netherlands; 2 Center for Environmental Health Research, National Institute for Public Health and the Environment, Bilthoven, the Netherlands; 3 Department of Internal Medicine and Laboratory for Clinical Thrombosis and Haemostasis, Cardiovascular Research Institute Maastricht (CARIM), Maastricht University, Maastricht, the Netherlands

**Keywords:** air pollution, blood coagulation, cardiovascular disease, inflammation, platelet aggregation

## Abstract

**Background:**

Air pollution has consistently been associated with increased morbidity and mortality due to respiratory and cardiovascular disease. Underlying biological mechanisms are not entirely clear, and hemostasis and inflammation are suggested to be involved.

**Objectives:**

Our aim was to study the association of the variation in local concentrations of airborne particulate matter (PM) with aerodynamic diameter < 10 μm, carbon monoxide, nitrogen monoxide, nitrogen dioxide, and ozone with platelet aggregation, thrombin generation, fibrinogen, and C-reactive protein (CRP) levels in healthy individuals.

**Methods:**

From 40 healthy volunteers, we collected 13 consecutive blood samples within a 1-year period and measured light-transmittance platelet aggregometry, thrombin generation, fibrinogen, and CRP. We performed regression analysis using generalized additive models to study the association between the hemostatic and inflammatory variables, and local environmental concentrations of air pollutants for time lags within 24 hr before blood sampling or 24–96 hr before blood sampling.

**Results:**

In general, air pollutants were associated with platelet aggregation [average, +8% per interquartile range (IQR), *p* < 0.01] and thrombin generation (average, +1% per IQR, *p* < 0.05). Platelet aggregation was not affected by *in vitro* incubation of plasma with PM. We observed no relationship between any of the air pollutants and fibrinogen or CRP levels.

**Conclusions:**

Air pollution increased platelet aggregation as well as coagulation activity but had no clear effect on systemic inflammation. These prothrombotic effects may partly explain the relationship between air pollution and the risk of ischemic cardiovascular disease.

Epidemiologic studies have linked elevated levels of both gaseous and (ultra-)fine particulate matter (PM) ambient air pollutants to increased morbidity and mortality due to respiratory and cardiovascular disease (McCreanor et al. 2007; Peng et al. 2008). Underlying biological mechanisms are unclear, but inflammation and hemostasis are suggested to be involved (Brook et al. 2004; Peters et al. 1997; Ruckerl et al. 2006). It has been postulated that inhaled gases, and also ultrafine PM because of its very small particle size (< 0.1 μm), can readily cross the lung epithelium into the bloodstream (Nemmar et al. 2002). There they can have direct, transient systemic effects leading to a prothrombotic state, such as enhanced platelet activation and thrombin generation (Nemmar et al. 2004; Radomski et al. 2005; Seaton et al. 1995). In contrast, larger particles that cannot pass the alveolar–blood barrier will perturb the lung epithelium, where they may give rise to local inflammation (Donaldson et al. 2001; Salvi et al. 1999). Under experimental conditions, human or animal exposure to a controlled high dose of air pollutants has been shown to cause pulmonary inflammation that leads to a systemic release of cytokines. This in turn induces *de novo* synthesis of inflammatory biomarkers in the liver, such as fibrinogen, which also plays a major part in blood clotting, and C-reactive protein (CRP). In general, at least 24 hr elapses from the onset of this protein synthesis to a clear increase in plasma levels of inflammatory markers (Ruckerl et al. 2007). We therefore hypothesized that air pollution has both direct and indirect effects on platelet aggregation and coagulation, but only indirect effects on plasma levels of the inflammatory variables fibrinogen and CRP.

Previous studies have aimed primarily at finding epidemiologic associations between concentrations of air pollution and health effects, including mortality, or associations between experimentally controlled exposures to air pollution and various biological variables in human and animal models (Brook 2007; Gong 1992). However, studies are still lacking that focus on the effect of air pollution on hemostasis and inflammation in a real-life urban situation over a longer period of time, especially because continuous long-term monitoring shows large variations within each year in local concentrations of air pollutants (Figure 1) (Brook et al. 2004). Therefore, the aim of this study was to investigate longitudinally (i.e., repeatedly over 1 year) the associations between local urban concentrations of ambient air pollution and plasma markers of hemostasis and inflammation. In addition, we aimed to investigate *in vitro* whether PM can have an effect on platelet aggregation.

## Materials and Methods

### Study population

Between January 2005 and December 2006, we included 40 healthy individuals who were living or working in the city center of Rotterdam, the Netherlands, a city agglomerate with almost 1 million inhabitants. Exclusion criteria were symptoms of chronic infectious diseases, acute infections, or any surgical procedure within the preceding 3 months. We collected from each participant blood at 11–13 (mean, 12.5) different visits throughout a 1-year period. In total, we collected 498 blood samples on 197 days. For each subject, the minimal interval between successive blood collections was 3 days and the maximal interval was 6 months, with a similar pattern of distribution in each subject. We collected data on demographics and cardiovascular risk factors using a standardized questionnaire. To minimize the effect of circadian variation on plasma levels of biomarkers, we took blood samples between 0900 and 1100 hours. The study protocol is in accordance with the Declaration of Helsinki and was approved by the Medical Ethics Committee of the Erasmus University Medical Center. We obtained written informed consent from each participant.

### Air pollution monitoring data

We obtained concentrations of PM with aerodynamic diameter < 10 μm (PM_10_), carbon monoxide, nitrogen monoxide, nitrogen dioxide, and ozone from the Dutch National Air Quality Monitoring Network (National Institute for Public Health and the Environment 2009), which measured these air pollutants hourly at monitoring station no. 418 (Schiedamse Vest, Rotterdam, the Netherlands). is monitoring site is located in the Rotterdam city center and is subject to frequent quality control to ensure its ability to represent urban background air pollution. For data analysis, we calculated 6-hr means and then combined them into 12-hr and 24-hr means. If more than two hourly concentrations were missing for a 6-hr mean, we imputed them using data from five other monitoring stations of the Dutch National Air Quality Monitoring Network that were all within 25 km of Rotterdam.

### Blood collection

Blood was drawn by venipuncture in the antecubital vein using the Vacutainer system (Becton Dickinson, Plymouth, UK) containing sodium citrate (final concentration, 3.2%). Plasma was obtained by centrifugation at 1,500*g* for 10 min at 4°C and stored in aliquots at −80°C until further analysis. For platelet aggregation, blood was centrifuged at 150*g* for 15 min to obtain platelet-rich plasma (PRP) and subsequently at 1,500*g* for 10 min to obtain platelet-poor plasma (PPP). We adjusted PRP with autologous PPP to 200 × 10^9^ platelets/L (P200), which was used in platelet aggregation experiments.

### Laboratory measurements

#### Light-transmittance platelet aggregometry

We performed adenosine diphosphate (ADP)–induced light-transmittance platelet aggregometry as described previously (van Gestel et al. 2003). We chose ADP as the agonist because the ADP pathway in platelets plays an important role in atherothrombosis (Woulfe et al. 2001). We preincubated P200 with aspirin (100 μmol/L final) for 20 min and brought it to a physiologic calcium concentration of 16.6 mM by adding calcium chloride (CaCl_2_) (Merck & Co., New York, NY, USA) after preincubation with the thrombin-inhibitor -phenylalanyl-L -prolyl-L-arginine chloromethyl ketone (40 μmol/L, final; Merck & Co., Darmstadt, Germany). We induced platelet aggregation by 5 and 2.5 μmol/L ADP (Sigma Chemical Co., St. Louis, MO, USA) and determined maximal aggregation and late aggregation (residual aggregation at 6 min after the maximum representing platelet aggregate stability) by recording for 10 min on a four-channel optical aggregometer (Chrono-log, Kordia Life Sciences, Leiden, the Netherlands). Because of logistic reasons, platelet aggregation could be performed in only a subset of 139 plasma samples from 16 individuals.

We studied direct *in vitro* effects of PM on platelet aggregation by adding different types of diluted PM (reference PM with diameter size < 0.1, 2.5, or 10 μm, diesel soot collected with a diesel generator, urban background dust collected from a local baghouse filter extract, or EHC-93 reference dust that was collected in Ottawa, Ontario, Canada) to P200 or whole blood in various concentrations (range, 0–100 μg/mL) for different incubation periods (range, 0–2 hr) and performing ADP-induced light-transmittance or ADP-induced impedance whole-blood platelet aggregation experiments (Chronolog), respectively. We compared results with those obtained with aliquot samples without incubation with PM.

#### Thrombin generation

We measured thrombin generation in tissue factor (TF)-triggered PPP with the calibrated automated thrombogram (CAT) method (rombinoscope, Maastricht, the Netherlands) (Hemker et al. 2003). We conducted measurements on 80 μL plasma with final concentrations of 1 and 5 pM TF (PPP reagent low and PPP reagent; rombinoscope) and 4 μM phospholipids. We obtained thrombin calibrator from Thrombinoscope. We read fluorescence in a Fluoroskan Ascent reader (Thermo Labsystems OY, Helsinki, Finland) equipped with a 390/460-nm filter set. We calculated thrombin generation curves with the rombinoscope software. We derived three parameters from the thrombin generation curves: lag time (defined as the time to reach one-sixth of the peak height), endogenous thrombin potential (ETP), and peak height. A thrombin generation curve is characterized by the initial burst of thrombin formation and the lag time, which depends on the amount of TF present in the sample or added to the plasma to trigger coagulation. Furthermore, the lag time is negatively associated with the plasma levels of factors VII and IX, antithrombin, free protein S, and free TF pathway inhibitor (Dielis et al. 2008). The other two main parameters, ETP and peak height, reflect the potential of plasma to generate thrombin and have been suggested to indicate a state of hypercoagulability when elevated (ten Cate-Hoek et al. 2008). Both the ETP and peak height are determined by plasma levels of fibrinogen, factor XII, antithrombin, and free TF pathway inhibitor (Dielis et al. 2008).

#### Fibrinogen and CRP

We determined fibrinogen levels according to von Clauss (Instrumentation Laboratory, IJsselstein, the Netherlands) and the prothrombin (PT)-derived method (Dade Thrombin Reagent, Siemens Diagnostics, Leusden, the Netherlands) on a Sysmex CA-1500 automated coagulation analyzer (Siemens Diagnostics, Leusden, the Netherlands). We measured CRP levels by means of an in-house high-sensitivity ELISA with polyclonal rat anti-human CRP antibodies (Dako, Glostrup, Denmark) and a CRP calibrator (Dako).

### Statistical analysis

We present data as mean ± SD for continuous variables and as counts and percentages for categorical variables. We performed linear regression analysis between plasma levels and air pollution concentration at different periods before each blood sampling. We analyzed data in R software (version 2.5.1; R Foundation for Statistical Computing, Vienna, Austria) using generalized additive models with individual intercepts for each subject, day of the week as a an indicator variable, and penalized spline smoothers for date (to adjust for trend and seasonality) and meteorologic parameters (temperature, pressure, and relative humidity). We used the software to optimize degrees of freedom used for the splines, according to the procedure described by Wood (2001). We used 10 knots as a starting point. The effective degrees of freedom for trend ranged from 1 to 8 for the different models.

A time lag corresponds to a mean concentration of an air pollutant that we calculated from concentrations hourly measured within the corresponding time window preceding each blood sampling, for which we set the time of the blood sampling to 0 hours. Time lags represent direct effects D_0–6_, D_0–12_, and D_0–24_; indirect effects I_24–48_, I_48–72_, and I_72–96_; and both direct and indirect effects D+I_0–96_ (Figure 2). In addition, for O_3_ we added the maximum concentration that we measured within the 24 hr preceding each blood sampling to study the effect of peak exposures. The longitudinal study design included repeated measures analysis, whereby subjects served as their own references. In this analysis, plasma levels in 13 blood samples of each subject were associated with the corresponding local concentrations of air pollutants. We normalized effects of air pollution on plasma variables and present them as percent change of the variable of interest for one interquartile range (IQR) of an air pollutant (%/IQR) (+ indicates an increase of the %/IQR; − indicates a decrease). In this model, effects can be compared among all air pollutants and all plasma variables. We considered a two-sided value of *p* < 0.05 statistically significant. For CRP and fibrinogen, we determined only indirect effects of air pollution (time lags I_24–48_, I_48–72_, and I_72–96_) (Ruckerl et al. 2007). We also performed all analyses after excluding smokers (*n* = 7) or women using oral contraceptives (*n* = 9). We calculated the correlation coefficients between the concentrations of different air pollutants by means of Pearson’s correlation test.

## Results

### Study population and concentration profiles of air pollutants

We included 40 healthy subjects with a mean age of 41 years in the study (Table 1). Twenty-six women participated in the study (65% of total). In total, there were seven current smokers (18%).

**Figure 1:**
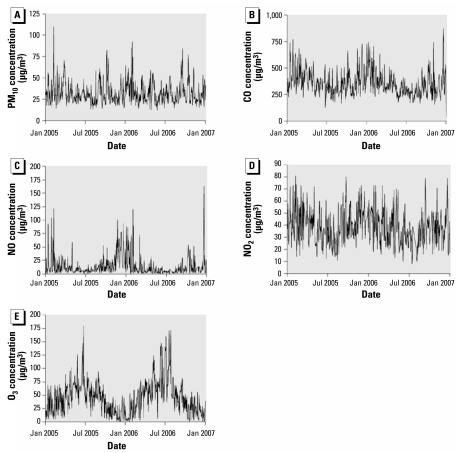
Concentration profiles of air pollutants during the study period: 24-hr mean concentrations for PM_10_ (*A*), CO (*B*), NO (*C*), and NO_2_ (*D*) and 8-hr mean concentrations (1200 to 2000 hours) for O_3_ (*E*).

**Figure 2:**
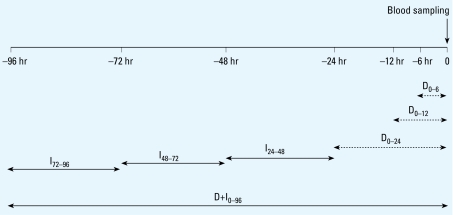
Time lags of estimated exposure to air pollution before blood sampling. The time of each blood sampling was set to 0 hours. Time lags represent means of air pollution concentrations that were determined hourly within the corresponding time window preceding each blood sampling. Dashed arrows represent direct effects of air pollution (D_0–6_, D_0–12_, and D_0–24_), and solid arrows indirect effects (I_24–48_, I_48–72_, and I_72–96_). Time lag D+I_0–96_ represents the mean concentration within 4 days before blood sampling.

The profile of air pollution concentrations throughout the study period shows quite variable levels of air pollutants (Table 2, Figure 1). The correlation coefficients between the concentrations of different air pollutants were >0.6 per each of the studied time lags and were negative between O_3_ and the other air pollutants (−0.4 to −0.6).

### Platelet aggregation

**Figure 3:**
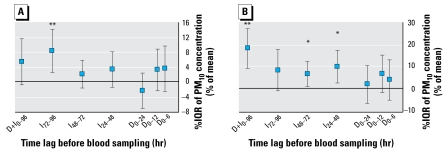
Effects of PM_10_ on platelet aggregation: estimated effects of PM_10_ on maximal aggregation (*A*) and late aggregation (*B*) as percent change from the mean of each individual per IQR increase in PM_10_ concentration. Time lags D_0–6_, D_0–12_, and D_0–24_ represent direct effects of PM_10_ on platelet aggregation, and time lags I_24–48_, I_48–72_, and I_72–96_, indirect effects. Time lag D+I_0–96_ represents the effect of 4-day mean concentration of PM_10_ on platelet aggregation. *p < 0.05, **p < 0.01.

The characteristics of the subset of subjects in whom platelet aggregation was performed (*n* = 16) were similar to those of the remaining 24 subjects, except that there were no users of oral contraceptives in this subset of 16 subjects (data not shown). We observed indirect effects of air pollution on platelet aggregation, represented by a positive significant association between 5 μmol/L ADP-induced maximal aggregation and PM_10_ concentrations for time lag I_72–96_ (+8%/IQR, *p* < 0.01) (Figure 3A). In addition, late aggregation was significantly associated with PM_10_ for time lags I_24–48_ and I_48–72_ (+10 and +6%/IQR, respectively; both *p* < 0.05) and for time lag D+I_0–96_ (+18%/IQR, *p* < 0.01) (Figure 3B). We also observed significant associations between maximal aggregation and CO, NO, and NO_2_ for time lag I_48–72_ (+8, +6, and +6%/IQR, respectively; all *p* < 0.01) and for time lag D+I_0–96_ (+9%/IQR of CO, *p* < 0.05, and +8%/IQR of NO, *p* < 0.01) (Table 3). Similarly, late aggregation was significantly associated with CO, NO, and NO_2_ for these time lags (I48–72: +18, +8, and +9%/IQR, respectively; D+I0–96: +20, +13, and +16 %/IQR, respectively; all *p* < 0.01) (Table 3). In addition, we observed a significant association between O_3_ during time lag I48–72 and maximal daily concentration of O_3_ and late aggregation (−26 and −16%/IQR, respectively, both *p* < 0.05) (Table 3). We obtained similar results when we induced platelet aggregation with 2.5 μmol/L ADP, instead of 5 μmol/L ADP, and when we performed the analyses in nonsmokers only or after exclusion of women using oral contraceptives.

We observed no direct effect of PM_10_ on platelet aggregation, because we noted no association between the PM_10_ concentration and maximum platelet aggregation or late aggregation for the direct-effect time lags D_0–6_, D_0–12_, and D_0–24_ (Figure 3). We confirmed this absence of direct effects *in vitro*, because the addition of various types of PM to P200 or whole blood did not lead to any changes in light transmittance or impedance whole-blood platelet aggregation, compared with an aliquot P200 sample to which we added no PM. However, a direct *in vivo* effect was suggested for CO, because we observed a significant positive association with late aggregation (+11, +12, and +11%/IQR for D_0–6_, D_0–12_, and D_0–24_, respectively; all *p* < 0.05) (Table 3).

### Thrombin generation

**Figure 4:**
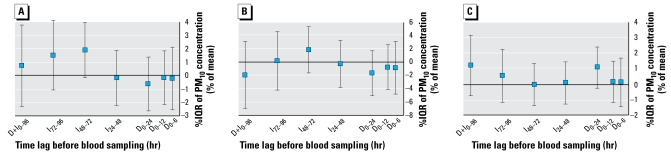
Effects of PM_10_ on thrombin generation: estimated effects of PM_10_ on ETP (*A*), peak height (*B*), and lag time (*C*) as percent change from the mean for each individual per interquartile increase in PM_10_ concentration. Time lags D_0–6_, D_0–12_, and D_0–24_ represent direct effects of PM_10_ on thrombin generation, and time lags I_24–48_, I_48–72_, and I_72–96_, indirect effects. Time lag D+I_0–96_ represents the effect of 4-day mean concentration of PM_10_ on thrombin generation. *p < 0.05.

We observed a significant increase in ETP for the gaseous pollutants CO, NO, and NO_2_ at time lags representing indirect effects of air pollution (I_24–48_, +2%/IQR of NO and +4%/IQR of NO_2_; I_72–96_, +3%/IQR of CO and +2%/IQR of NO; all *p* < 0.05) and a significant increase in peak thrombin generation (I_24–48_, +4%/IQR of NO and +8%/IQR of NO_2_, both *p* < 0.01; I_72–96_, +4%/IQR of NO, *p* < 0.05) (Table 4). In addition, peak thrombin generation was significantly increased by 6% per IQR of maximal daily concentration of O_3_ (Table 4). The associations with PM_10_ levels were less clear and not statistically significant, although the estimates for time lags that represent indirect effects on ETP were mainly positive (Figure 4A). The lag time of thrombin generation was significantly lower when the concentrations of gaseous pollutants were increased at time lags representing indirect effects (I_24–48_, −2%/IQR of NO and −3%/IQR of NO_2_, both *p* < 0.01; I_48–72_, −2%/IQR of NO_2_, *p* < 0.05; I_72–96_, −1%/IQR of NO, *p* < 0.05), except for the time lag D_0–24_ (+2%/IQR of CO, *p* < 0.05) (Table 4). We observed no clear associations between PM_10_ and peak height or lag time of thrombin generation (Figure 4B,C). The associations between air pollutants and parameters of thrombin generation induced by 5 pM TF showed similar associations (data not shown). We also obtained similar results for thrombin generation parameters when we performed the analyses in nonsmokers only or after excluding women using oral contraceptives (data not shown).

### Inflammation

**Figure 5:**
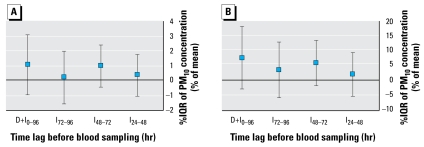
Indirect effects of PM_10_ on inflammation: estimated indirect effects of PM_10_ on fibrinogen (*A*) and CRP (*B* ) as percent change from the mean of each individual per interquartile increase in PM_10_ concentration. Time lags I_24–48_, I_48–72_, and I_72–96_ represent indirect effects of PM_10_ on inflammation. Time lag D+I_0–96_ represents the effect of 4-day mean concentration of PM_10_ on inflammation. *p < 0.05.

Our data suggest that there are no indirect effects of PM_10_ on inflammation, because we found no statistically significant associations between PM_10_ concentrations and either CRP or fibrinogen levels (Figure 5). We obtained similar results for CO, NO, NO_2_, and O_3_ (Table 5) and when we performed the analyses in nonsmokers only or after excluding women using oral contraceptives (data not shown).

## Discussion

The main observation of this study was that air pollution (except O_3_) was associated with increased platelet aggregation and increased thrombin generation, but not with the inflammatory markers fibrinogen and CRP. The significant associations between various air pollutants and increased maximal aggregation and late aggregation for time lags within 48–96 hr before blood sampling suggest that exposure to air pollution indirectly increases blood thrombogenicity. These indirect effects may be the result of air pollution–induced synthesis of TF (Sun et al. 2008), which can increase *in vivo* platelet reactivity (Kuijpers et al. 2008). TF-bearing microparticles have also been suggested to contribute to these effects on platelets either directly, or indirectly via increased blood coagulability (Morel et al. 2006). Another possible mechanism of indirect platelet activation might reside in pulmonary oxidative stress and the activation of subsets of white blood cells that lead to a systemic lowering of endothelial- and platelet-derived nitrogen oxide and concomitant platelet activation (Brook 2008). Our results also indicate that it is unlikely that blood platelets are directly activated by contact with artificial surfaces of PM because we observed no association between platelet aggregation and PM concentrations for time lags representing direct effects. We confirmed these findings with our *in vitro* experiments, in which we saw no direct effects (0–2 hr) of the addition of PM on platelet aggregation.

Results for thrombin generation suggest that air pollution leads to an overall tendency toward a hypercoagulable state, because both ETP and peak thrombin generation were increased after exposure to higher levels of gaseous air pollutants. Again, these indirect effects on thrombin generation may be caused by elevated levels of TF. Notably, only gaseous pollutants, and not PM_10_, were associated with these indirect effects. The gaseous air pollutants, especially NO_2_ and CO, can be considered markers for motor vehicle traffic and have been shown to be highly correlated with ultrafine particles (Cyrys et al. 2003). It is mainly this subset of ultrafine particles from the overall PM air pollution that has an effect on thrombin generation (Spronk HM, ten Cate H, unpublished data). is could explain why only the gaseous pollutants, and not PM_10_ mass concentration, was associated with thrombin generation in this study. Another mechanism may involve altered synthesis of pro- and anticoagulant proteins. However, previous studies on air pollution–related changes in plasma levels of PT, factor VII, antithrombin, protein C, and protein S are conflicting (Baccarelli et al. 2007; Pekkanen et al. 2000; Ruckerl et al. 2006; Seaton et al. 1999). In our study, changes in thrombin generation were independent of the changes in fibrinogen concentrations. Because thrombin generation depends not only on fibrinogen but also on other pro- and anticoagulant proteins, this suggests that air pollution can induce changes in hemostatic balance that are not inflammation driven.

In our study, air pollution was not associated with systemic inflammation. We measured fibrinogen and CRP, two sensitive inflammatory markers that are consistently associated with cardiovascular risk. The results from epidemiologic studies on air pollution and inflammation are conflicting, as are those from laboratory studies on the inflammatory responses in human volunteers or animals after experimentally controlled exposure to air pollution (Baccarelli et al. 2007; Chuang et al. 2007; Maier et al. 2008; Nemmar et al. 2003; Seaton et al. 1999). Because fibrinogen and CRP do not reflect all aspects of inflammation, measuring other inflammatory markers, such as interleukin 6 and tumor necrosis factor-α, may still reveal associations between air pollution and inflammation (Mutlu et al. 2007; Ruckerl et al. 2006).

Most of the significant associations observed in the present study concern more than one air pollutant, especially in the case of late aggregation, which was associated with all studied air pollutants. The correlation coefficients between the concentrations of air pollutants were moderate and mainly positive (> 0.6), except between O_3_ and the other air pollutants (−0.4 to −0.6). This probably explains the observed opposite effects of O_3_ on most of the plasma variables, compared with the effects of PM_10_, CO, NO, and NO_2_. However, when we analyzed these effects of O_3_ in a two-pollutant model with PM_10_, only the effects of PM_10_ remained statistically significant with similar estimates (data not shown), suggesting that PM_10_, rather than O_3_, is responsible for the observed effects on platelet aggregation and thrombin generation. This was in contrast to the results of the two-pollutant models that combined PM_10_ with CO, NO, or NO_2_, which indicated that effects of these gases are mainly independent of the effects of PM_10_ (data not shown). Nevertheless, it remains difficult to completely discern causal air pollutant(s) from their surrogate markers.

The present study was designed to represent a real-life urban situation. Our approach combines several strengths. First, we have evaluated the effects of air pollution on blood parameters over a period of 1 year. In this longitudinal study design, participants were their own controls, which ensures the most reliable estimates of acute effects of exposure to air pollution. Second, we selected subjects with a similar exposure to local air pollution. Third, several studies have shown that central site measurements correlate well with personal exposures for longitudinal acute effects (Janssen et al. 1998, 2005; World Health Organization 2006). Although the study participants have spent a significant amount of time living or working in Rotterdam during the study period, their estimated exposure to air pollution may vary. However, we did not design our study to correct for these possible variations. Another limitation of the study is that the PM_10_ mass concentration is a poor measure of its biological activity, because the corresponding particles are heterogeneous in composition (e.g., endotoxins and metals) and may therefore trigger different biological responses (Brook et al. 2004). All subjects in this study were healthy volunteers. The observed effects of air pollution on hemostasis may differ (being possibly more pronounced) in subjects with coronary artery disease or subjects at higher risk for this disease.

In conclusion, our data show a significant association between exposure to air pollution and systemic prothrombotic tendency of the blood via increased platelet aggregation and thrombin generation in a healthy population. This association may point to a relevant biological mechanism that contributes to the risk association between air pollution and cardiovascular disease.

## Figures and Tables

**Table 1:** Characteristics of the study population (*n* = 40).

Characteristic	Value
Age (years)	41 ± 15
No. of females	26 (65)
Body mass index (kg/m^2^)	22.6 ± 2.0
No. of smokers	7 (18)
No. of oral contraceptive users	9 (23)
Blood parameters	
Fibrinogen (g/L)	2.6 ± 0.5
CRP (mg/L)	0.6 ± 1.2
Platelet aggregation (*n* = 16)	
Maximal aggregation (%)	65 ± 13
Late aggregation (%)	46 ± 20
Thrombin generation	
ETP (nM/min)	999 ± 317
Peak (nM)	141 ± 71
Lag time (min)	4.2 ± 0.9

Values are no. (%) for categorical variables and mean ± SD for continuous variables.

**Table 2:** Concentrations (μg/m^3^) of air pollutants during the study period.

Air pollutant	Median	25th–75th percentile	Maximum
PM_10_	29.3	23.8–39.2	110.1
CO	333	276–412	1,283
NO	7	4–15	163
NO_2_	37	27–48	81
O_3_	44	21–63	180

**Table 3:** Estimated changes of platelet aggregation parameters associated with mean air pollutant levels at various time lags before blood sampling.

	Air pollutant				
Time lag	PM_10_	CO	NO	NO_2_	O_3_
Maximal platelet aggregation					

D_0–6_	3.5 (−2.5 to 9.6)	−3.6 (−9.3 to 2.1)	1.3 (−4.4 to 7.1)	−2.3 (−7.3 to 2.7)	7.0 (−1.7 to 15.7)
D_0–12_	3.2 (−2.4 to 8.8)	−4.7 (−11.0 to 1.5)	0.7 (−5.4 to 6.8)	−2.6 (−8.4 to 3.3)	4.1 (−4.6 to 12.8)
D_0–24_	−2.5 (−7.2 to 2.3)	−2.6 (−7.9 to 2.7)	1.9 (−3.0 to 6.9)	−3.0 (−10.3 to 4.3)	4.9 (−6.6 to 16.3)
I_24–48_	3.3 (−1.5 to 8.1)	−1.1 (−7.2 to 4.9)	1.2 (−4.1 to 6.5)	−0.6 (−6.6 to 5.3)	−5.7 (−20.3 to 9.0)
I_48–72_	2.0 (−1.7 to 5.8)	8.4 (2.5 to 14.3)**	6.1 (2.4 to 9.7)**	5.6 (1.5 to 9.7)**	−8.1 (−18.8 to 2.7)
I_72–96_	8.3 (2.5 to 14.1)**	−0.1 (−5.1 to 5.0)	−0.4 (−5.5 to 4.8)	1.2 (−4.5 to 6.9)	−1.6 (−10.4 to 7.3)
D+I_0–96_	5.4 (−0.8 to 11.6)	9.5 (1.6 to 17.4)*	8.5 (2.8 to 14.1)**	3.0 (−3.8 to 9.8)	−7.2 (−22.4 to 8.1)
Maximum					1.1 (−9.5 to 11.7)

Late aggregation					

D_0–6_	3.7 (−5.4 to 12.9)	10.5 (0.8 to 20.3)*	8.1 (−1.2 to 17.3)	3.3 (−5.3 to 11.8)	−15.0 (−30.4 to 0.5)
D_0–12_	6.6 (−2.0 to 15.1)	11.6 (1.2 to 21.9)*	8.5 (−0.7 to 17.6)	7.5 (−2.3 to 17.2)	−14.1 (−29.0 to 0.8)
D_0–24_	1.7 (−6.8 to 10.2)	11.2 (1.4 to 21.0)*	8.9 (1.12 to 16.6)*	9.9 (−2.5 to 22.3)	−17.3 (−35.2 to 0.6)
I_24–48_	9.8 (2.4 to 17.2)*	7.5 (−2.2 to 17.1)	5.5 (−1.5 to 12.4)	1.9 (−9.0 to 12.7)	−18.4 (−39.0 to 2.2)
I_48–72_	6.4 (0.7 to 12.2)*	18.1 (8.4 to 27.8)**	7.9 (2.3 to 13.4)**	8.9 (2.6 to 15.2)**	−26.0 (−44.1 to −7.8)**
I_72–96_	8.2 (−1.2 to 17.6)	4.2 (−5.5 to 13.9)	3.4 (−5.7 to 12.6)	4.8 (−4.3 to 13.9)	1.2 (−14.8 to 17.3)
D+I_0–96_	18.1 (9.1 to 27.1)**	20.4 (8.4 to 32.4)**	13.0 (4.9 to 21.1)**	16.1 (5.0 to 27.2)**	−17.1 (−40.8 to 6.7)
Maximum					−16.4 (−31.0 to −1.8)*

Data are percent change of 5 μmol/L ADP-induced maximal platelet aggregation and late aggregation (6 min after maximum), with 95% confidence intervals in parentheses. Values are based on hourly measurements from a monitor located within the city center of Rotterdam. Effects for time lags are presented for all air pollutants; for O_3_ we additionally present the effect of the maximum concentration in the 24 hr preceding blood sampling. Blood was drawn from all subjects between 0900 and 1100 hours.

* *p* < 0.05;

** *p* < 0.01.

**Table 4:** Estimated changes of thrombin generation associated with mean air pollutant levels at various time lags before blood sampling.

	Air pollutant				
Time lag	PM_10_	CO	NO	NO_2_	O_3_
ETP					

D_0–6_	−0.2 (−2.6 to 2.1)	−1.5 (−3.7 to 0.8)	−0.4 (−2.3 to 1.5)	−1.2 (−3.6 to 1.2)	3.2 (−0.3 to 6.7)
D_0–12_	−0.2 (−2.2 to 1.8)	−1.1 (−3.4 to 1.1)	−0.4 (−2.0 to 1.1)	−0.2 (−2.8 to 2.4)	0.8 (−2.8 to 4.4)
D_0–24_	−0.7 (−2.7 to 1.4)	−1.5 (−3.9 to 0.9)	−0.3 (−2.2 to 1.6)	0.3 (−2.5 to 3.1)	0.3 (−3.8 to 4.5)
I_24–48_	−0.2 (−2.3 to 1.9)	−0.7 (−3.4 to 2.0)	1.9 (0.1 to 3.7)*	3.5 (0.2 to 6.8)*	−0.9 (−5.4 to 3.6)
I_48–72_	1.9 (−0.2 to 4.0)	0.8 (−1.9 to 3.4)	0.8 (−0.8 to 2.5)	2.1 (−1.0 to 5.2)	−0.6 (−4.5 to 3.3)
I_72–96_	1.5 (−1.1 to 4.2)	3.5 (0.8 to 6.2)*	2.1 (0.2 to 4.0)*	0.8 (−1.9 to 3.5)	−0.6 (−4.1 to 3.0)
D+I_0–96_	0.7 (−2.3 to 3.8)	0.8 (−2.7 to 4.3)	1.7 (−1.1 to 4.5)	1.1 (−1.7 to 4.0)	1.0 (−3.2 to 5.2)
Maximum					2.3 (−1.2 to 5.8)

Peak					

D_0–6_	−0.8 (−4.8 to 3.1)	−2.5 (−6.3 to 1.3)	−0.4 (−3.6 to 2.8)	−1.5 (−5.5 to 2.5)	5.7 (−0.2 to 11.7)
D_0–12_	−0.7 (−4.1 to 2.6)	−1.9 (−5.7 to 1.9)	−0.4 (−3.1 to 2.3)	−0.7 (−5.1 to 3.7)	2.8 (−3.4 to 8.9)
D_0–24_	−1.6 (−5.0 to 1.8)	−3.3 (−7.3 to 0.7)	−0.6 (−3.9 to 2.6)	−0.6 (−5.3 to 4.1)	2.6 (−4.8 to 9.9)
I_24–48_	−0.2 (−3.7 to 3.3)	−1.3 (−6.1 to 3.6)	4.1 (1.1 to 7.2)**	8.0 (2.4 to 13.6)**	0.2 (−7.3 to 7.8)
I_48–72_	1.9 (−1.6 to 5.4)	−0.5 (−5.0 to 4.0)	1.2 (−1.6 to 4.0)	3.7 (−1.5 to 9.0)	1.4 (−5.1 to 8.0)
I_72–96_	0.2 (−4.2 to 4.6)	3.8 (−0.8 to 8.4)	3.5 (0.4 to 6.7)*	−0.2 (−4.8 to 4.4)	2.6 (−3.3 to 8.5)
D+I_0–96_	−1.9 (−6.9 to 3.2)	−1.7 (−7.5 to 4.2)	3.1 (−1.7 to 7.8)	1.0 (−4.1 to 6.0)	6.6 (−0.7 to 13.8)
Maximum					6.3 (0.3 to 12.3)*

Lag time					

D_0–6_	0.1 (−1.4 to 1.7)	1.0 (−0.5 to 2.5)	−0.1 (−1.4 to 1.1)	0.0 (−1.6 to 1.6)	−0.3 (−2.6 to 2.0)
D_0–12_	0.2 (−1.2 to 1.5)	1.0 (−0.5 to 2.5)	0.0 (−1.1 to 1.0)	0.0 (−1.8 to 1.7)	−0.4 (−2.7 to 2.0)
D_0–24_	1.1 (−0.2 to 2.4)	1.6 (0.1 to 3.1)*	0.6 (−0.6 to 1.8)	0.2 (−1.6 to 2.0)	−0.3 (−2.9 to 2.3)
I_24–48_	0.1 (−1.3 to 1.5)	0.4 (−1.3 to 2.2)	−1.8 (−2.9 to −0.7)**	−3.1 (−5.1 to −1.0)**	3.0 (0.4 to 5.7)*
I_48–72_	0.0 (−1.4 to 1.4)	−1.0 (−2.7 to 0.7)	−0.8 (−1.8 to 0.3)	−2.5 (−4.3 to −0.6)*	1.2 (−1.1 to 3.6)
I_72–96_	0.6 (−1.2 to 2.3)	−1.5 (−3.2 to 0.2)	−1.4 (−2.6 to −0.2)*	0.0 (−1.8 to 1.7)	0.6 (−1.6 to 2.8)
D+I_0–96_	1.2 (−0.7 to 3.2)	0.1 (−2.1 to 2.2)	−1.1 (−2.8 to 0.5)	−0.7 (−2.5 to 1.1)	0.6 (−2.0 to 3.2)
Maximum					−1.2 (−3.5 to 1.0)

Percent change of thrombin with their 95% confidence intervals indicated in parentheses. Thrombin generation was induced with 1 pM TF and 4 μmol/L phospholipids. Values are based on hourly measurements from a monitor located within the city center of Rotterdam. Effects for time lags are presented for all air pollutants; for O_3_ we additionally present the effect of the maximum concentration in the 24 hr preceding blood sampling. Blood was drawn from subjects between 0900 and 1100 hours.

* *p* < 0.05;

** *p* < 0.01.

**Table 5:** Estimated changes of inflammatory markers associated with mean air pollutant level time lags representing indirect effects before blood samplings.

	Air pollutant				
Time lag	PM_10_	CO	NO	NO_2_	O_3_
Fibrinogen					

I_24–48_	0.4 (−1.1, 1.8)	0.0 (−1.7, 1.8)	0.1 (−1.0, 1.3)	0.4 (−1.7, 2.5)	−0.6 (−3.2, 2.1)
I_48–72_	1.0 (−0.5, 2.4)	0.0 (−1.8, 1.9)	0.3 (−0.8, 1.4)	1.4 (−0.6, 3.4)	−1.4 (−3.8, 1.0)
I_72–96_	0.2 (−1.6, 2.0)	−0.1 (−1.9, 1.7)	0.1 (−1.1, 1.4)	−0.4 (−2.3, 1.4)	0.5 (−1.7, 2.8)

CRP					

I_24–48_	1.9 (−5.6, 9.4)	3.2 (−6.4, 12.8)	3.6 (−2.9, 10.0)	6.5 (−4.9, 17.8)	−0.5 (−14.7, 13.8)
I_48–72_	5.8 (−2.0, 13.5)	−1.9 (−12.5, 8.7)	0.1 (−6.5, 6.7)	−0.1 (−11.0, 10.8)	3.7 (−9.7, 17.2)
I_72–96_	3.4 (−6.2, 12.9)	−4.5 (−15.3, 6.3)	−4.6 (−12.0, 2.9)	−6.9 (−17.2, 3.5)	5.9 (−6.8, 18.7)

Percent change with 95% confidence intervals indicated in parentheses. Values are based on hourly measurements from a monitor located within the city center of Rotterdam. Blood was drawn from all subjects between 0900 and 1100 hours.

## References

[b1-ehp-117-995] Baccarelli A, Zanobetti A, Martinelli I, Grillo P, Hou L, Giacomini S (2007). Effects of exposure to air pollution on blood coagulation. J Thromb Haemost.

[b2-ehp-117-995] Brook RD (2007). Is air pollution a cause of cardiovascular disease? Updated review and controversies. Rev Environ Health.

[b3-ehp-117-995] Brook RD (2008). Air pollution: what is bad for the arteries might be bad for the veins. Arch Intern Med.

[b4-ehp-117-995] Brook RD, Franklin B, Cascio W, Hong Y, Howard G, Lipsett M (2004). Air pollution and cardiovascular disease: a statement for healthcare professionals from the Expert Panel on Population and Prevention Science of the American Heart Association. Circulation.

[b5-ehp-117-995] Chuang KJ, Chan CC, Su TC, Lee CT, Tang CS (2007). The effect of urban air pollution on inflammation, oxidative stress, coagulation, and autonomic dysfunction in young adults. Am J Respir Crit Care Med.

[b6-ehp-117-995] Cyrys J, Stolzel M, Heinrich J, Kreyling WG, Menzel N, Wittmaack K (2003). Elemental composition and sources of fine and ultrafine ambient particles in Erfurt, Germany. Sci Total Environ.

[b7-ehp-117-995] Dielis AW, Castoldi E, Spronk HM, van Oerle R, Hamulyak K, Ten Cate H (2008). Coagulation factors and the protein C system as determinants of thrombin generation in a normal population. J Thromb Haemost.

[b8-ehp-117-995] Donaldson K, Stone V, Seaton A, MacNee W (2001). Ambient particle inhalation and the cardiovascular system: potential mechanisms. Environ Health Perspect.

[b9-ehp-117-995] Gong H (1992). Health effects of air pollution. A review of clinical studies. Clin Chest Med.

[b10-ehp-117-995] Hemker HC, Giesen P, Al Dieri R, Regnault V, de Smedt E, Wagenvoord R (2003). Calibrated automated thrombin generation measurement in clotting plasma. Pathophysiol Haemost Thromb.

[b11-ehp-117-995] Janssen NA, Hoek G, Brunekreef B, Harssema H, Mensink I, Zuidhof A (1998). Personal sampling of particles in adults: relation among personal, indoor, and outdoor air concentrations. Am J Epidemiol.

[b12-ehp-117-995] Janssen NA, Lanki T, Hoek G, Vallius M, de Hartog JJ, Van Grieken R (2005). Associations between ambient, personal, and indoor exposure to fine particulate matter constituents in Dutch and Finnish panels of cardiovascular patients. Occup Environ Med.

[b13-ehp-117-995] Kuijpers MJ, Munnix IC, Cosemans JM, Vlijmen BV, Reutelingsperger CP, Egbrink MO (2008). Key role of platelet procoagulant activity in tissue factor-and collagen-dependent thrombus formation in arterioles and venules *in vivo* differential sensitivity to thrombin inhibition. Microcirculation.

[b14-ehp-117-995] Maier KL, Alessandrini F, Beck-Speier I, Hofer TP, Diabate S, Bitterle E (2008). Health effects of ambient particulate matter—biological mechanisms and inflammatory responses to *in vitro* and *in vivo* particle exposures. Inhal Toxicol.

[b15-ehp-117-995] McCreanor J, Cullinan P, Nieuwenhuijsen MJ, Stewart-Evans J, Malliarou E, Jarup L (2007). Respiratory effects of exposure to diesel traffic in persons with asthma. N Engl J Med.

[b16-ehp-117-995] Morel O, Toti F, Hugel B, Bakouboula B, Camoin-Jau L, Dignat-George F (2006). Procoagulant microparticles: disrupting the vascular homeostasis equation?. Arterioscler Thromb Vasc Biol.

[b17-ehp-117-995] Mutlu GM, Green D, Bellmeyer A, Baker CM, Burgess Z, Rajamannan N (2007). Ambient particulate matter accelerates coagulation via an IL-6-dependent pathway. J Clin Invest.

[b18-ehp-117-995] National Institute for Public Health and the Environment (RIVM) (2009). Milieuportaal voor professionals.

[b19-ehp-117-995] Nemmar A, Hoet PH, Vanquickenborne B, Dinsdale D, Thomeer M, Hoylaerts MF (2002). Passage of inhaled particles into the blood circulation in humans. Circulation.

[b20-ehp-117-995] Nemmar A, Hoylaerts MF, Hoet PH, Nemery B (2004). Possible mechanisms of the cardiovascular effects of inhaled particles: systemic translocation and prothrombotic effects. Toxicol Lett.

[b21-ehp-117-995] Nemmar A, Nemery B, Hoet PH, Vermylen J, Hoylaerts MF (2003). Pulmonary inflammation and thrombogenicity caused by diesel particles in hamsters: role of histamine. Am J Respir Crit Care Med.

[b22-ehp-117-995] Pekkanen J, Brunner EJ, Anderson HR, Tiittanen P, Atkinson RW (2000). Daily concentrations of air pollution and plasma fibrinogen in London. Occup Environ Med.

[b23-ehp-117-995] Peng RD, Chang HH, Bell ML, McDermott A, Zeger SL, Samet JM (2008). Coarse particulate matter air pollution and hospital admissions for cardiovascular and respiratory diseases among Medicare patients. JAMA.

[b24-ehp-117-995] Peters A, Doring A, Wichmann HE, Koenig W (1997). Increased plasma viscosity during an air pollution episode: a link to mortality?. Lancet.

[b25-ehp-117-995] Radomski A, Jurasz P, Alonso-Escolano D, Drews M, Morandi M, Malinski T (2005). Nanoparticle-induced platelet aggregation and vascular thrombosis. Br J Pharmacol.

[b26-ehp-117-995] Ruckerl R, Greven S, Ljungman P, Aalto P, Antoniades C, Bellander T (2007). Air pollution and inflammation (interleukin-6, C-reactive protein, fibrinogen) in myocardial infarction survivors. Environ Health Perspect.

[b27-ehp-117-995] Ruckerl R, Ibald-Mulli A, Koenig W, Schneider A, Woelke G, Cyrys J (2006). Air pollution and markers of inflammation and coagulation in patients with coronary heart disease. Am J Respir Crit Care Med.

[b28-ehp-117-995] Salvi S, Blomberg A, Rudell B, Kelly F, Sandstrom T, Holgate ST (1999). Acute inflammatory responses in the airways and peripheral blood after short-term exposure to diesel exhaust in healthy human volunteers. Am J Respir Crit Care Med.

[b29-ehp-117-995] Seaton A, MacNee W, Donaldson K, Godden D (1995). Particulate air pollution and acute health effects. Lancet.

[b30-ehp-117-995] Seaton A, Soutar A, Crawford V, Elton R, McNerlan S, Cherrie J (1999). Particulate air pollution and the blood. Thorax.

[b31-ehp-117-995] Sun Q, Yue P, Kirk RI, Wang A, Moatti D, Jin X (2008). Ambient air particulate matter exposure and tissue factor expression in atherosclerosis. Inhal Toxicol.

[b32-ehp-117-995] ten Cate-Hoek AJ, Dielis AW, Spronk HM, van Oerle R, Hamulyak K, Prins MH (2008). Thrombin generation in patients after acute deep-vein thrombosis. Thromb Haemost.

[b33-ehp-117-995] van Gestel MA, Heemskerk JW, Slaaf DW, Heijnen VV, Reneman RS, oude Egbrink MG (2003). *In vivo* blockade of platelet ADP receptor P2Y12 reduces embolus and thrombus formation but not thrombus stability. Arterioscler Thromb Vasc Biol.

[b34-ehp-117-995] Wood SN (2001). GAMs and generalized ridge regression for R. R News.

[b35-ehp-117-995] World Health Organization (2006). Particulate matter, ozone, nitrogen dioxide and sulfur dioxide. Air Quality Guidelines: Global Update.

[b36-ehp-117-995] Woulfe D, Yang J, Brass L (2001). ADP and platelets: the end of the beginning. J Clin Invest.

